# Understanding Rates of Marijuana Use and Consequences Among Adolescents in a Changing Legal Landscape

**DOI:** 10.1007/s40429-017-0170-y

**Published:** 2017-10-05

**Authors:** Elizabeth J. D’Amico, Joan S. Tucker, Eric R. Pedersen, Regina A. Shih

**Affiliations:** 1 RAND Corporation, 1776 Main St., Santa Monica, CA 90407-2138 USA; 2 RAND Corporation, 1200 South Hayes St., Arlington, VA 22202-5050 USA

**Keywords:** Marijuana, Medical marijuana, Adolescents, Legislation, Cannabis use disorder, Prevention, Intervention

## Abstract

**Purpose of Review:**

There is not one answer to address whether marijuana use has increased, decreased, or stayed the same given changes in state legalization of medical and non-medical marijuana in the USA.

**Recent Findings:**

Evidence suggests some health benefits for medical marijuana; however, initiation of marijuana use is a risk factor for developing problem cannabis use. Though use rates have remained stable over recent years, about one in three 10th graders report marijuana use, most adolescents do not view the drug as harmful, and over 650,000 youth aged 12 to 17 struggle with cannabis use disorder.

**Summary:**

Although the health benefits of medical marijuana are becoming better understood, more research is needed. Intervention and prevention programs must better address effects of marijuana, acknowledging that while there may be some benefits medically, marijuana use can affect functioning during adolescence when the brain is still developing.

## Introduction

Since 1996, when California became the first state to pass a comprehensive medical marijuana law (MML), 29 states in the USA have legalized marijuana for medical purposes as of 2017. Eight states have expanded marijuana laws that allow for legalized recreational, production, and for-profit sales among adults aged 21 and older. Washington DC also has legalized marijuana possession for recreational purposes, but not production or sales. It has been speculated that more states will begin passing recreational marijuana laws in the coming elections. The current paper provides a brief overview of marijuana use and consequences among adolescents given the changing legal landscape in the USA. We first discuss overall trends of marijuana use and consequences from use. We next address the benefits and harms associated with the use of medical marijuana given the current state of research. Third, we provide a brief review of the evidence regarding effects of MMLs on perceptions of risk, use, and consequences. Finally, we discuss the challenge of reducing marijuana use among adolescents and policy implications for prevention and intervention for this age group.

According to one of the biggest national studies, Monitoring the Future [1], rates of lifetime marijuana use among adolescents over the last 5 years have remained fairly steady among 12th graders (45% report lifetime use); whereas among younger ages, initiation of use has declined somewhat. For example, in 2012, about 16% of eighth graders had initiated marijuana use, but in 2016, 13% reported initiation of the drug. Likewise, about 35% of 10th graders reported lifetime use of marijuana in 2012, which decreased to 30% in 2016 [1]. Despite these decreases in lifetime use, it is important to note that these percentages still reflect a significant number of teens that report trying marijuana (e.g., one in three 15–16-year-olds). In addition, another large national study, the National Survey on Drug Use and Health (NSDUH), estimated that approximately 650,000 youth aged 12 to 17 met criteria for cannabis use disorder (CUD) in 2015 [2].

Problematic marijuana use continues to be an issue for young people. One recent study by D’Amico and colleagues [3•] examined rates of both marijuana and alcohol use, as well as alcohol use and cannabis use disorders in a large and diverse sample of 1573 youth age 12–18 (21% Black, 51% Hispanic) attending a primary care appointment in either California or Pennsylvania. Past-year marijuana use was slightly lower than alcohol use (37% and 42%, respectively), as was past-year heavy marijuana use (e.g., using two or more times in 1 day; 19%) compared to heavy alcohol use (e.g., five or more drinks; 22%); however, CUD was three times more prevalent than alcohol use disorder (14% and 4%, respectively). Thus, although rates of use for alcohol and marijuana were fairly similar among these adolescents, they were more likely to report problematic use of marijuana [3•]. For example, on the Diagnostic Interview Schedule for Children Version IV (DISC-IV), which included updated DSM-5 criteria for CUD, 48% of youth reported that they had tried to quit or cut down their marijuana use compared to 16% of adolescents who reported that they tried to quit or cut back on their drinking. In addition, 32% of teens in this study reported going to school or work when they were high or smoking marijuana while at school or work compared to 14% who reported going to school or work after drinking or drinking while at school or work. Other problems youth reported included smoking more marijuana than they thought they would (40%), getting into arguments with family members or friends because of using marijuana (23%), and that marijuana caused them to get sad, depressed, or irritable (14%).

There is growing evidence that marijuana use may cause more problems in functioning during adolescence than alcohol use. One 2016 large longitudinal school-based study found that marijuana use was associated with poorer functioning in high school across more domains compared to alcohol use [4]. Specifically, our team examined how marijuana and alcohol use trajectories from age 11 to 17 years were associated with key domains of functioning during high school. Teens with greater marijuana use indicated more academic unpreparedness and poorer academic performance, increased delinquency, and worse mental health in high school. Youth that reported higher alcohol use also indicated poorer functioning, but only in two domains: greater academic unpreparedness and delinquency. Furthermore, non-white youth appeared to be disproportionately affected by marijuana use (as well as alcohol use), reporting worse outcomes for academics and health compared to white youth, even at the same levels of use [4]. Overall, research documents that adolescent marijuana use and resulting consequences are a public health concern that need to be addressed.

## Medical Marijuana: Benefits and Harms

The health benefits of medical marijuana are becoming better understood, though there is still much research to be done, and the majority of work in this area has been established with adults. A recent report from the National Academies of Sciences, Engineering, and Medicine [5] concluded that there is moderate to substantial evidence supporting marijuana use as an effective treatment for chronic pain, alleviating chemotherapy-induced nausea and vomiting, improving spasticity symptoms among patients with multiple sclerosis, and improving short-term sleep outcomes among those with obstructive sleep apnea syndrome. Importantly, however, the same report concluded that there is substantial evidence that marijuana use has negative long-term effects such as worsening respiratory symptoms (e.g., chronic cough, bronchitis), increased risk of motor vehicle accidents when driving under the influence, lower birth rates of offspring from mothers who use the drug, and increased risk for developing schizophrenia or other psychoses [5]. Overall, it is important to note that studies are typically limited by a lack of standardization of dosing and potency, including cannabidiol (CBD) to tetrahydrocannabinol (THC) ratios, which makes marijuana a challenging substance to regulate for medical purposes [6].

The report from the National Academies of Sciences, Engineering, and Medicine [5] also concludes that there is substantial evidence that early initiation of marijuana use, as well as increases in frequency of use during adolescence, are risk factors for the development of problem cannabis use later in life. Other earlier reviews of adolescent marijuana use have concluded that early and chronic use may have negative effects on several cognitive and mental health factors, such as executive functioning [7], depression [8], and use of other substances [9]. Thus, even though some benefits of medical marijuana have been found, as noted above, most of the research on medical marijuana to date has been conducted with adults. Overall, very little is known about medicinal benefits of marijuana for adolescents. Indeed, the available research on harms suggests that early initiation of marijuana, increased marijuana use during adolescence, and chronic marijuana use over time is linked to problems.

Concerning the use of medical marijuana by adolescents specifically, Boyd and colleagues [10] found that only 1% of the approximately 4400 12th graders in the 2012/2013 Monitoring the Future sample reported using medical marijuana that they had obtained as part of their own recommendation from a provider that qualified them for participation in their state’s medical marijuana program. (This is often called a marijuana “prescription” by those that obtain this recommendation; thus, hereafter we refer to this as a prescription.) Interestingly, 6% of the sample reported use of medical marijuana that was obtained from someone else’s prescription (diverted marijuana use) [10]. Of the 12th graders that reported past-year marijuana use, 80% did *not* obtain it from a legal or medical source, 3% reported marijuana use from their own prescription, and 17% reported “diverted marijuana use.” Findings indicated that those who obtained marijuana from their own prescription or from someone else’s prescription were more at risk across a host of outcomes, including higher rates of frequent and daily marijuana use, greater likelihood of reporting “being hooked” on marijuana, and greater risk for using other prescription drugs non-medically and using other illicit drugs than those who obtained marijuana from a non-legal or non-medical source [10]. Thus, adolescents who obtain medical marijuana with a prescription, either their own or someone else’s, represent a group that is at high risk for numerous problems.

## Effects of Medical Marijuana Laws on Perceptions of Risk, Use, and Consequences

Given the increasingly widespread legalization of medical and recreational marijuana across the USA, there have been a number of recent high-quality epidemiological studies examining changes in overall marijuana use rates among adolescents before and after the passage of marijuana legalization laws attempting to answer the following important question: have marijuana use rates increased, decreased, or stayed the same following legalization? At this point, it is difficult to determine the “final answer.” [11, 12] This is partly due to the heterogeneous nature of these studies. For example, some studies are national, some occur in single states with legalized medical marijuana, and still others take place in states where marijuana is legal for both medical and recreational possession, sale, and cultivation [13]. The story is further complicated by the nuances in policy in different states (e.g., registration requirements, home cultivation, dispensaries) and the timing of these policies [14]. For example, Pacula and colleagues [14] demonstrated the disadvantages of treating medical MMLs generically, showing that specific modes of regulation differentially influenced consumption, highlighting the importance of understanding the heterogeneity of these laws. Specifically, they found that access to dispensaries or home cultivation may increase marijuana consumption, including among adolescents, even though simple dichotomous indicators (e.g., yes MML versus no MML) were generally not associated with marijuana use. In addition, they found that marijuana dependence was higher in states that had more lenient access to medical marijuana, such as home cultivation and state acceptance of dispensaries [14].

Other studies have also failed to find a clear link between MMLs and increased use among adolescents [12, 15], including one recent large scale study of over one million adolescents surveyed between 1991 and 2014. Results showed that despite finding higher rates of marijuana prevalence in states that had passed an MML compared to those that did not, rates of marijuana use did not increase significantly within states from before to after the passage of MMLs [16•]. In addition, although according to NSDUH the rates of CUDs have been declining among youth over the past 12 years [2], rates of CUD among adults have increased in states with MMLs [17]. Pacula and Smart [18] note, however, that disparate findings across and within studies could also be attributed to the way that marijuana use is measured, such as whether the studies examining associations between MMLs and adolescent marijuana use utilized measures of past-month use, frequency of use, quantity of use, heavy use, or dependence.

With the rapidly changing landscape of marijuana policy across the USA, there has been increasing interest in assessing the effects of these policy changes on teens as the outcomes may not be clear for some time [11]. For example, Friese and Grube [19] examined the association between adolescent marijuana use and voter approval of medical marijuana and the number of medical marijuana cards issued in a sample of 17,482 adolescents age 13–19 across several counties in Montana. They found that youth reported greater lifetime and past 30-day use of marijuana when they lived in counties with a higher percentage of voters approving legalization of medical marijuana; however, the number of medical marijuana cards was not related to marijuana use [19]. This suggests that more positive perceptions of the drug may be affecting overall adolescent use.

Overall, there has been a trend towards more positive views of marijuana among both adults [20] and teens in recent years [21, 22]. More than 50% of 10th and 12th graders across the USA now endorse the belief that *smoking* marijuana regularly does not carry great risk (note that this question does not address other ways of using marijuana, such as vaping or edibles) [23]. In Washington state, which legalized medical and non-medical marijuana in 2012 (with stores commencing sale of recreational marijuana in 2014), one study found that the positive association between low perceived harm and marijuana use has grown stronger since 2000 [22]. A 2014 Monitoring the Future study cross-sectionally examined perceived harmfulness of marijuana use by grade, stratified by state MML status, and found that overall, adolescents living in states that had ever passed an MML were less likely to perceive marijuana as harmful [24].

Many of these positive beliefs for marijuana may come from social media and/or advertising, which has increased as MMLs have passed. For example, among people ages 17 to 19 years, the popular pro-marijuana Twitter handle @stillblazingtho was in the top 10% of all Twitter handles followed [25]. Examinations of the more proximal effects of MML passage are critical: advertising, accessibility, and the growing prevalence of adults who use medical marijuana may drive adolescent perceptions of use. In a cohort of approximately 8000 youth with a mean age of 13, D’Amico and colleagues [26] found that sixth and eighth graders’ exposure to advertising for medical marijuana was associated with both intentions to use marijuana and marijuana use 1 year later. This highlights the importance of beginning to think about regulations for marijuana advertising [26], similar to regulations that are in place for tobacco and alcohol [27].

Though billboards, magazines, and social media can increase young people’s exposure to marijuana advertisements, the proliferation of medical marijuana and recreational marijuana dispensaries no doubt also increases adolescents’ exposure to the drug. Specific methods to examine accessibility to dispensaries have been proposed to map dispensary locations given that these tend to fluctuate (e.g., a dispensary that is open today may not be open in 6 months) [28–30]. More work will be needed as policies rapidly change to get a better handle on effects of the actual dispensaries, including longitudinal studies that can address temporality. To date, only one study has examined how proximity to marijuana dispensaries affect adolescent marijuana use. This cross-sectional study used Monitoring the Future data and found that the availability of medical marijuana dispensaries within a 5-mile buffer zone was associated with a higher likelihood of recent marijuana use by eighth graders, and being within either a 5-mile or 25-mile buffer zone was associated with an increased likelihood of recent marijuana use for 10th graders [31]. Monitoring the Future data [1] indicate that 35% of eighth graders and the majority of 10th (64%) and 12th (81%) graders report that marijuana is “fairly easy” or “very easy” to get. More work is needed in this area to understand the pathways through which proximity to dispensaries may be related to subsequent marijuana use among adolescents.

In terms of adverse consequences related to MML passage, Plunk and colleagues examined the effects of exposure to MMLs on high school completion, college enrollment, and college completion [32]. They used data from the 2000 Census and 2001–2014 American Community Surveys. Exposure was defined as any exposure to policy of generic MMLs (i.e., irrespective of specific features of MML policy) while adolescents were of high school age (i.e., 14–18). They also assigned policy exposure based on the number of years that youth were exposed to the MML between the ages of 14 and 18 with possible values of 0–4 to reflect years of exposure during high school (i.e., exposure beginning at age 18 would be 1 year, age 17 was equal to 2 years, age 16 was equivalent to 3 years, and ages 14–15 equaled 4 years). They found that MML exposure was associated with a 0.40 increase in the probability of not earning a high school diploma (from 3.99% probability of not earning a HS diploma to 4.39%). In addition, exposure to MML during high school was associated with a higher probability of both college non-enrollment and degree non-completion (a 0.85 increase from 45.30% to 46.15%). Furthermore, MML exposure was associated with an increase in daily marijuana use among 12th graders (up from 1.25% to 2.11%) [32].

Other health risks may be related to changes in marijuana legalization. There is evidence that marijuana legalization is associated with the co-use of tobacco and marijuana. Data from the 2013 National Survey on Drug Use and Health (NSDUH) indicate that a higher proportion of past 30-day tobacco and marijuana co-users reside in states where medical marijuana is legal compared to states where it is illegal [33]. Although the reasons for marijuana and tobacco co-use are poorly understood and likely multifaceted [34, 35], there has been increasing public health concern that tobacco use may begin to increase among young people as a consequence of marijuana legalization. Cannabis and tobacco are often smoked on the same occasion, with some early research suggesting that these simultaneous users are at greater risk for CUDs [36]. Co-administration is another popular form of co-use; an example of this is blunt smoking, which involves replacing some or most of the tobacco in a cigar with marijuana [37].

Furthermore, marijuana-impaired driving has doubled in recent years for high school seniors across the USA, and teens report driving under the influence of marijuana at higher rates than driving under the influence of alcohol [38]. Nearly one in five teens reports driving under the influence of marijuana, 34% of whom believed their driving ability was improved after marijuana use [39], and younger drivers are especially likely to believe that driving under the influence of marijuana is socially acceptable and safe [40]. These data suggest that youth are not as concerned about driving under the influence of marijuana compared to alcohol, emphasizing that marijuana use and consequences may be viewed differently than alcohol use and consequences [41]. Overall, findings highlight the importance of addressing marijuana use and its potential consequences among this population.

## The Challenge of Reducing Marijuana Use Among Adolescents

It is crucial that we begin to address marijuana use in this changing legal landscape. This requires a good understanding of how medical marijuana may be used and a focus on recreational use. The National Academies of Sciences, Engineering, and Medicine [5] report discusses identifying research gaps, improving data collection, and proposing strategies to address barriers for marijuana research [5]. Furthermore, this report noted that marijuana potency has increased [42], and that different forms of marijuana have become popular, including edibles, vaping, and dabs [43•, 44]. In order to work with youth around this issue, providers must have a good understanding of the research, the reasons that youth may use marijuana (e.g., for a perceived health benefit versus recreationally), and the ways in which they may use marijuana.

Our prevention and intervention work with at risk teens [45] and emerging young adults age 18–25 [46] has shown that marijuana use may be more difficult to change than alcohol use, in part because youth view the consequences from marijuana use differently [41, 46]. Specifically, they tend to see fewer consequences occurring from their marijuana use because they view it as safer to use than alcohol, and they are also able to more clearly connect consequences to their drinking behavior than to their marijuana use [41]. Given this, and recent evidence of perceptions of marijuana harm decreasing [22, 25], intervention and prevention programs must better address the effects of marijuana, acknowledging that while there may be some benefits medically, marijuana use can affect functioning during adolescence [3, 4] when the brain is still developing [47].

Our work with at risk youth in a teen court setting [41, 48] and with urban Native American adolescents [49], for example, has emphasized the importance of discussing both the pros and cons of marijuana use with youth using a motivational interviewing (MI) approach [50]. In this MI approach, therapists discuss the nuances of research on the medical “benefits” of marijuana use versus recreational use, and also address how frequency and quantity of use and marijuana use over time can impact health, relationships, and attainment of educational and work goals. We have created a website at www.groupmiforteens.org that provides free online training for conducting MI in groups with teens and shows how to best discuss marijuana use, including addressing medical marijuana use. We also make our manuals available for free on this site, which provide different ways to talk to teens about marijuana use in a non-confrontational and collaborative manner.

## Policy Implications and Conclusions

In sum, work has shown that specific components of MMLs (e.g., access to dispensaries or home cultivation) are associated with marijuana use and that these same components are associated with fatal car accidents and increased heavy drinking [14]. In addition, exposure to medical marijuana advertising [26] and perhaps proximity to dispensaries [31] are both associated with an increased likelihood of marijuana use among younger adolescents, although no longitudinal studies have yet been conducted on proximity to dispensaries. History from the tobacco and alcohol industries emphasizes the importance of having policies and regulations around advertising and outlet density. Pacula and colleagues [27] highlight specific areas that policymakers may want to address regarding marijuana legalization including: developing regulations that help reduce access, availability, and use by adolescents, driving under the influence, and concurrent use of marijuana and alcohol, particularly in public places. As other states move to legalize marijuana for both medical and recreational purposes, it will be crucial to address how marijuana should be regulated in order to decrease the chances of harm occurring.
